# On the potential for saturated buffers in northwest Ohio to remediate nutrients from agricultural runoff

**DOI:** 10.7717/peerj.9007

**Published:** 2020-04-21

**Authors:** Stephen J. Jacquemin, Greg McGlinch, Theresa Dirksen, Angela Clayton

**Affiliations:** 1 Agriculture and Water Quality Education Center, Wright State University - Lake Campus, Celina, OH, USA; 2 Department of Horticulture and Crop Science, The Ohio State University, Columbus, OH, USA; 3 Mercer County Community and Economic Development Office, Celina, OH, USA

**Keywords:** Saturated buffers, Nutrient manangement, Harmful algal blooms, Nutrient tile runoff, Edge of field, Best management practice

## Abstract

Nutrient loading from nonpoint source runoff in the Midwest has emerged as one of the largest threats to water quality as the frequency of harmful algal blooms, hypoxic zones, and issues associated with human-resource interactions have risen abruptly over the past several decades. In this study, a saturated buffer ~500 m in length located in the western basin of the Lake Erie watershed was evaluated for its potential to reduce edge of field runoff and nutrient loading. Saturated buffers reduce runoff by routing subsurface tile drainage water into the riparian zone, providing an opportunity for drainage volume as well as nutrient reduction of runoff waters. Over a 12-month study period, controlled drainage was used to redirect nearly 25% of the total tile flow into the riparian zone from a subwatershed in corn/soybean rotation with near complete reductions of dissolved nitrogen and phosphorus from tile inflows averaging 4.7 and 0.08 mg/L, respectively, as well as total reduction of suspended sediments (average 10.4 mg/L). This study provides additional evidence that riparian areas are an important part of nutrient reduction strategies as they can act as both controlled drainage points by raising water tables in fields as well as nutrient sinks which couple to help mitigate nutrient runoff in the region.

## Introduction

Eutrophication of rivers and lakes as a result of nutrient loading has become one of the most widespread threats to freshwater ecosystems in the eastern United States. Excess nutrient loading has manifested on a region wide scale in the formation of harmful algal blooms (HABs) in lakes and slow-moving rivers where a variety of cyanobacteria genera (e.g., *Microcystis, Anabaena, Aphanizomenon, Planktothrix*, etc.) have negatively affected water clarity, esthetic, human health as a result of consumption or exposure to a variety of bloom produced toxins, as well as other aquatic life through formation of ‘dead zones’ (Braig et al., 2011; Brooks et al., 2016). Watersheds in the state of Ohio, such as the western basin of Lake Erie or Grand Lake St. Marys, have been particularly prone to the effects of eutrophication as a combined and often complicated relationship between nutrient runoff and watershed land use patterns, land management regimes, lake bathymetry, flow regime alterations, and climate change (Jarvie et al., 2017; Smith, King & Williams, 2015). And while HABs in the Great Lakes region are not novel, in recent years they have become increasingly frequent and severe (Michalak et al., 2013; Kane et al., 2014) with wide reaching economic and ecosystem level consequences (Wolf, Georgic & Klaiber, 2017).

In the western basin of Lake Erie, well over 80% of nutrient loading comes from non-point source landscape runoff, where the majority of acres are agricultural (Wilson et al., 2019). Thus, in order to improve water quality in the region, there is a distinct need to reduce and/or mitigate nutrient rich non-point source runoff. A myriad of effective edge of field practices (e.g., grass waterways, bioreactors, controlled drainage, etc.), management changes (e.g., cover crops, nutrient management plans, rules packages, etc.), and landscape level conservation efforts (e.g., wetlands) have been implemented and shown to benefit water quality in the northwest Ohio region (Jacquemin et al., 2018). However, despite these efforts, more needs to be done to mitigate runoff as current estimates from The Ohio Phosphorus Task Force outlined in the Great Lakes Water Quality Agreement suggest loading reductions of up to 40% (from 2008 loading data) in the western basin of Lake Erie are necessary to lessen HAB activity (USEPA Environment & Climate Change Canada, Annex 4 Objects & Targets Task Team, 2015).

To further reduce nutrient loading, a combination of expanding existing conservation practices as well as testing and implementing new approaches must be considered. Specific to northwest Ohio (and elsewhere in the Midwest), however, one of the most glaring challenges is the vast proliferation of subsurface tile drainage, which currently drain up to 80% of the landscape (Baker et al., 1975; Smith et al., 2015; Williams et al., 2016). While highly effective at rapidly draining water from the landscape in an agronomic context, these systems also quickly move nutrients away from fields and into aquatic systems by providing a conduit for nutrient loading in areas leading to tributary flow volumes often dominated by field runoff. Given that tile is engineered to move water quickly away from fields, rather than facilitating any kind of biogeochemical processes that may occur along the surface, runoff leaving field areas is often highly concentrated with nutrients (depending on field activities) and extremely flashy as it rapidly increases with precipitation where data from some studies from the Midwest find that 20+% of precipitation can be recovered in tile drainage (King, Fausey & Williams, 2014) until diminishing to low or no flow conditions following precipitation. Thus, solutions that specifically target tile runoff must be a high priority for nutrient reductions in the region.

One such best management practice that has garnered attention in recent years is the implementation of controlled tile drainage where tile water is held back in fields during appropriate agronomic times of the year using a system of stop logs placed at tile outflow points to hold back water volume in the field. Several recent studies have found 30+% loading reductions as a result of controlled drainage on individual tiles (Skaggs, Fausey & Evans, 2012; Williams, King & Fausey, 2015). Given the proliferation of tile drainage points in the region, it seems feasible to implement these structures and management plans on a wider basis to affect a more significant percentage of non-point runoff. It is important to recognize, however, that loading reductions identified as a result of controlled tile drainage are largely due to volume mitigation as a function of raising the water table, rather than nutrient processing of either nitrogen or phosphorus.

Extending controlled tile drainage by pairing with other conservation practices could provide a unique opportunity for enhancing the efficacy of these systems by capturing and processing nutrients from additional volume not held back in the field. Saturated buffers are an emerging best management practice that accomplishes this goal by rerouting a portion of the subsurface controlled tile drainage leaving the field through an existing riparian area by way of subsurface infiltration through the soil (Jaynes et al., 2018). Saturated buffers effectively utilize the drainage control box associated with existing controlled drainage to route overflow of water previously flowing unabated into streams into a network of subsurface tile extended into riparian zone areas. This system provides additional storage capacity of nutrient laden volumes by using the soils in the riparian area while also giving time for various biological mechanisms such as plant uptake, microbial utilization, and denitrification, as well as physical processes such as sedimentation or geochemistry attributed to reduce nitrogen, phosphorus, and sediment loading (Jaynes et al., 2018). And while riparian areas and their effect on surface waters are well studied, with biological and physical processes attributed to reductions of nitrogen, phosphorus, and sediment loading in streams (Hill, 1996; Dorioz et al., 2006; Hoffmann et al., 2009; Stutter, Chardon & Kronvang, 2012), far less is documented regarding their potential positive impact on subsurface drainage. As saturated buffers are a relatively new practice there is a paucity in the literature regarding exactly where they function best and what specific biological or physical mechanisms of action are primarily responsible for any possible nutrient reductions. The results of two recently published studies on the subject (Jaynes & Isenhart, 2014, 2019) are particularly encouraging, however, as it was found that saturated buffers can reduce loading from tile drainage by up to half by achieving near complete reduction of nitrogen through biological processes in the riparian area. And while these results are very encouraging, they are not specific to the landscape types or management common in northwest Ohio where little to no research has been undertaken on the effects of riparian infiltration on nutrient loading of both nitrogen and phosphorus. The kinds of simple tile flow processing and nutrient reduction data generated by basic studies such as these are essential pieces of information for future implementation in nutrient reduction strategies. Thus, the objective of this study was to begin to fill this gap in the literature in an effort to assess the potential for a saturated buffer located in a typical field in northwest Ohio to reduce runoff volume as well as to reduce nutrient concentrations as water moves through the subsurface riparian zone prior to entering into surface waters.

## Methods

**Figure 1:**
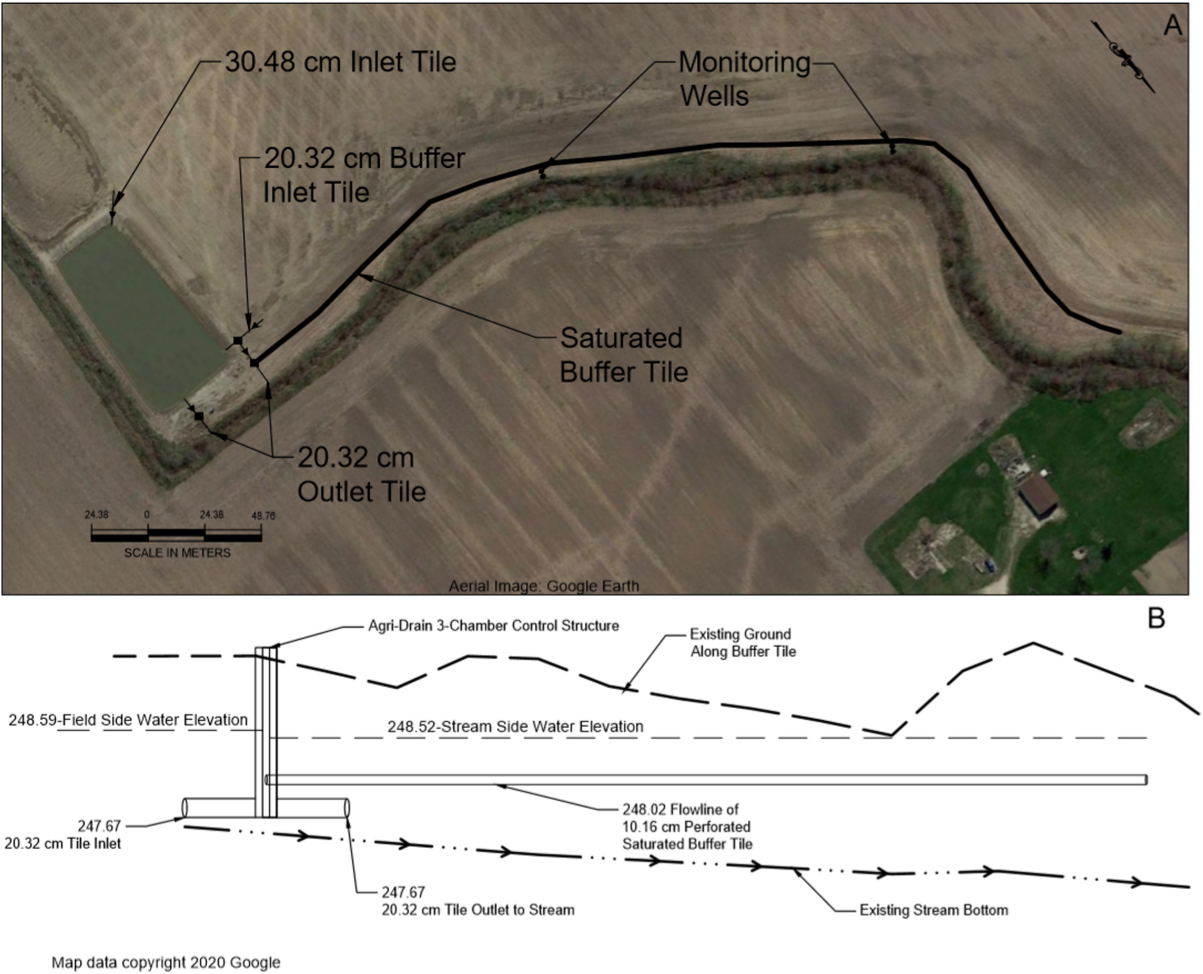
Site map (A) and overview of saturated buffer layout (B). Aerial image from Google Earth © 2020 Google.

The study field site was located on a privately owned and operated corn/soybean field (~55 hectares) in northwest Ohio (Mercer County, OH, USA). Prior to the start of the project, permission was obtained from the landowner and operator (Luke VanTilburg, VanTilburg Farms, Mercer County, Ohio) to access the site and monitor water quality associated with conservation practices for the duration of the project. Soils at the site (and larger region) are primarily comprised of poorly draining clays—specifically, Pewamo Silty Clay Loam (~60%) and Blount Silt Loam (~35%) with lesser percentages of Elliott Silt Loam and Glynwood Silt Loam (Priest, 1979). Soil composition, texture, and organic matter was verified at two points using a coring auger to collect samples in 150 mm intervals from the surface down to ~2.5 m. Soil organic matter was estimated based on gravimetric mass change following the loss-on-ignition methodology of Nelson & Sommers (1996). Given the predominance of poorly draining clay in the field soils, the site has been tiled with 100 mm perforated pipe spaced ~13 m on center to facilitate drainage. Ultimately, both surface and tile flow from the site drain into Dennison Ditch (Four Mile Creek) as part of the larger St. Marys River watershed before draining into the western basin of Lake Erie via the Maumee River. In 2015, Great Lakes Restoration Initiative funding was used to install several drainage water control practices, including a saturated buffer, on the site. The saturated buffer design included a 100 mm perforated pipe set at level grade bedded in coarse gravel approximately 1 m below ground level extending 500 m along the riparian zone. The buffer tile was connected to a 3-chambered drainage control structure and fed by a 150 mm tile inflow draining a 7.3 hectare subwatershed area within the field. The riparian zone was planted with an NRCS-CP-21 mix of warm-season grasses and forbs with an average vegetated width between field and stream of approximately 15 m. Elevation difference between the field and the riparian zone varied between 0.5 and 4 m. The field exhibited an average slope, based on LIDAR data, of approximately 1.4%. Groundwater monitoring wells consisted of 2.5 m sections of 5 cm diameter pipe and were positioned in pairs along two transects spaced at 1/3 and 2/3 distance between the buffer and stream following the expected path of groundwater flow spanning the bottom of the buffer tile to the flow line of the adjacent stream (Fig. 1). Monitoring well holes were excavated using a 30 cm augur to a depth of between 1.7 and 2.3 m consistent with the expected flow path of water following the hydrologic gradient from tile to adjacent stream bed. Once wells were situated they were backfilled with sand which surrounded the ~60 cm long section of well slots to allow water to percolate freely into the well with a thick layer of bentonite clay packed above this sand layer to prevent surface water from infiltrating well areas followed by a top soil layer sloped away from the wells to further reduce potential for surface infiltration down the well pipes. Following installation of the wells, the subsurface distribution pipe was flooded using an adjacent irrigation pond to verify water level change in wells, ensuring that they were in line with the expected infiltration path of water.

Monitoring of the saturated buffer included continuous estimation of tile flow volume into and out of the buffer control box, daily precipitation at the site, weekly groundwater level measurements, as well as weekly dissolved nutrient (nitrate-N and reactive P) and sediment concentrations from water samples whenever the buffer was actively charging. Flow volume was measured using a series of two depth loggers (HOBO Water Level Logger) placed in the first two chambers of the water control box. To facilitate water flow into the buffer, stop logs were set at 93 and 85 cm where the difference between the volume of water flowing over each (i.e., flow into the control box minus flow out of the control box, respectively), over time was interpreted as buffer infiltration rate. To improve accuracy and precision of flow estimates, V-notch weirs cut at 45-degree angles were used as control box boards instead of standard flat weirs. In addition, depth loggers were fixed inside 5 cm perforated PVC along the inside edge of the box to prevent movement in flow where they were kept free of debris and silt throughout the study. Depth measurements were logged in 30-min increments throughout the year and concatenated by mean daily flow. Depth loggers were tested for calibration on a biweekly basis. Water depth in control box chambers was translated to flow using the following equation, where *Q* is flow rate and *H* is water height above stop log notch, *Q* = 2.5866 × *H*^2.046^, noted in flow calculation engineering worksheets provided by the control box manufacturer (*Agri Drain*, https://www.agridrain.com). In the event that flow exceeded the V notch cut, width of the stop log (25.4 cm) was considered in calculations and amended to the total flow. Precipitation at the site was measured using a digital rain gauge logger (Rainwise RainLog Data Logger) calibrated in the laboratory with fixed water volumes and verified in the field with local weather information. Groundwater depth measurements taken on a weekly basis were done using a measuring rod placed into each well where the wetted level was recorded. Water samples for nutrient and sediment analyses were extracted from the field tile as well as from each of the four groundwater wells using a portable water pump. Once collected, a portion of each sample was filtered in the field using 0.45 μm syringe filters, prior to storing in a cooler for transport to the lab for analysis.

Water samples were analyzed for nitrate-N (NO_3_-N; dimethylphenol method), dissolved reactive phosphorus (ortho, DRP; ascorbic acid method), and total suspended solids (TSS; gravimetric method) following standard methods within 1 h of collection. Analytical detection limits for tests included minimums of 0.2 mg/L (NO_3_-N) and 0.025 mg/L (Ortho-P). When samples exceeded maximum ranges, dilutions were undertaken and samples were rerun. To ensure accuracy and precision of technique and analytical equipment, a combination of standards, spikes, duplicates (analytical and field), and outside lab verification for each parameter were undertaken with every sampling event.

## Results

### Soil descriptions

Soils in the field conformed to the clay-silt matrix consistent with overall site soil types; however, those in the riparian area were approximately 10% sandier than field soils, thus not agreeing with the overall Pewamo/Blount clay silty loam of the area. Additionally, organic matter percentages in the riparian were significantly higher than the field area, measuring 5.2% in the first meter of depth compared to 2.9%, respectively. These percentages decreased below 1 m, averaging 1.9% in the riparian compared with 1.7% in the field area when cored down to 2.5 m. The slight increase in sand, coupled with marked increase in organic content, likely served to improve lateral movement and infiltration rate of groundwater along with nutrient processing potential of the buffer.

### Precipitation and hydrology

**Figure 2:**
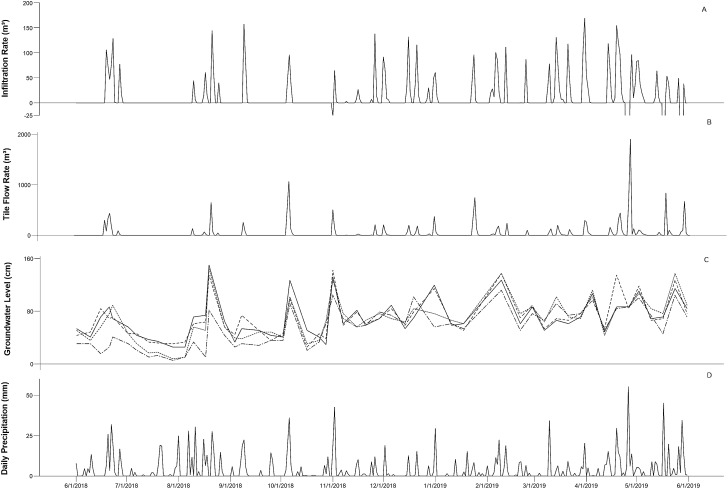
Overview of saturated buffer infiltration rate (A), tile flow (B), groundwater level (C), and daily precipitation (D).

Annual precipitation (June 2018 to May 2019) at the site was 1,430 mm resulting in a combined rainfall volume of 104,099 m^3^ (based on a watershed area of 7.3 hectares). This compares to annual averages from the site of approximately 1,049 mm, indicating a roughly 25% wetter than average year. Not surprisingly, spring rains represented the largest contributor to precipitation totals as both May and June exhibited among the highest monthly averages as well as the two highest single day precipitation totals ranging from 45 to 50+ mm. During this study period, tile inflow totaled 15,520 cubic meters, indicating that approximately 15% of the total precipitation volume drained through the tile. Tile flow was observed over 119 days of the year, and while surface flow was not measured, anecdotal observation of surface flow through one obvious lower swale did occur during the highest spring rains, indicating that the remaining 85% of precipitation was not all stored in soils. During this period, approximately 3,785 total cubic meters of flow was diverted into the buffer, indicating an infiltration annual average of ~25% of the total tile flow. This number varied substantially with precipitation totals and monitoring well water levels, reflecting a key relationship at this site between infiltration potential and ground saturation. Periods of highest groundwater levels saw the water table rise to 125+ cm in depth, occurring primarily during spring months which corresponded to an average infiltration rate of 20% and increased to between 35% and 44% during other times in the year. These lower spring infiltration rates included April and May of 2019, which exhibited mean infiltration rates of 4% and 0%, respectively. These periods of groundwater saturation created a small number of backflow events where the buffer actually discharged, reducing the overall efficacy of the system. Overall, the buffer actively charged 113 days, discharged 6 days, and did not receive any flow over 244 days of the year. During the 12-month study, greater than 50% of the total tile flow occurred over 10 days with flows ranging from 379 to 1,893 cubic meters per day. Not coincidentally, these periods corresponded to the highest precipitation and groundwater water levels and the time surrounding each of the backflow events (Fig. 2).

### Nutrient and sediment concentrations

**Figure 3:**
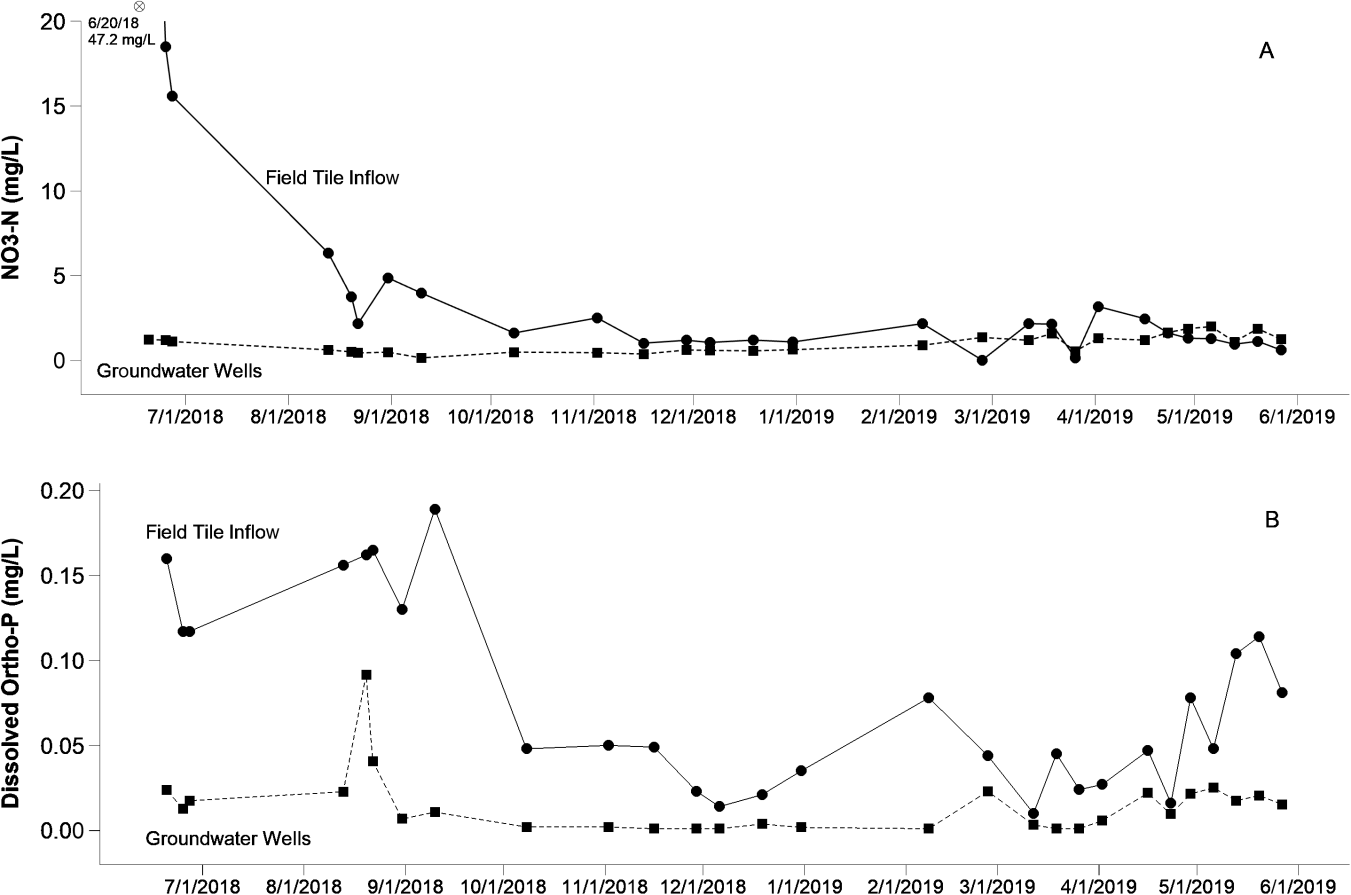
Nutrient variation of nitrate (A) and dissolved phosphorus (B) over time. The solid line indicates tile inflow sample, while the heavy dotted line signifies mean well concentrations across the four groundwater monitoring stations. Note that the asterisk in the nitrate-N plot signifies an outlier point that exceeds the scale bar, which has been truncated for clarity. Raw data for individual wells can be found in Appendix 1.

Nutrient and sediment sampling included 28 separate events where the highest nutrient values on a per sample basis were seen in the tile and first set of 2 wells leading away from the buffer, respectively (Fig. 3). Interestingly, the second well transect set further down the buffer routinely registered lower nutrient values when compared with the initial well transect closer to the control box. Given the positive pressure in the buffer tile maintained during charging events coupled with the high covariation of nutrients between field tile and wells, however, we do not interpret this as evidence of extensive surface water dilution. Furthermore, the presence of a thick blue and gray clay layers situated beneath the glacial till that comprise the parent material of area surface soils suggests that variations in monitoring well concentrations or levels are unlikely to have been influenced from subsurface upwellings from deep aquifers, etc. (Priest, 1979). Overall, both nutrients and sediment followed a similar pattern across the year long monitoring period where initial measurements taken during the spring and early summer seasons where highest and tapered off considerably throughout the year long study climbing slightly approaching the beginning of spring at the end of the study period (Fig. 3). This is undoubtedly a function of nutrient application from the producer where application records indicated nutrient addition schedules during the first part of year 1 but not yet occurring as the study concluded its monitoring period moving into the next agronomic year.

During the study period, NO_3_-N levels in drainage tile varied from <0.2 to 47.2 mg/L (average 4.7 mg/L; Fig. 3). Consistent and rapid declines in NO_3_-N concentrations were seen in well samples, which ranged from <0.2 to 1.98 mg/L (average 0.97 mg/L). Focusing just on those higher tile samples which registered above 2 mg/L of NO_3_-N, reduction efficiencies associated with the buffer averaged 75%. The only exception to this pattern was a single observation where nitrate was slightly higher in wells compared with field tile. We attribute this to variation in point estimates as a time lag between tile runoff and infiltration into the ground surrounding the wells. It is relevant to note that this exception only happened once and it occurred at a very low nitrate concentration. Similarly, comparatively high DRP levels in drainage tile, varying from <0.05 to 0.19 mg/L (average 0.08 mg/L), exhibited steep declines once infiltrated within the riparian buffer area, ranging from <0.05 to 0.09 mg/L (Fig. 3). Focusing just on those higher tile samples which registered above 0.05 mg/L of DRP, reduction efficiencies associated with the buffer averaged 80%. Specific to TSS values, drainage inflow values varied from 0.5 to 28.4 mg/L (average 10.4 mg/L). Since buffer water infiltrating through soils cannot carry particulate material, we interpret this sediment component as completely removed.

## Discussion

Over the study period, nearly 25% of subsurface flow was redirected into the buffer area where, upon infiltration, both nitrogen as well as dissolved phosphorus were consistently near or below minimum detectable levels. Based on these extremely low dissolved nutrient concentrations in the riparian buffer monitoring wells coupled with the additional area of subsurface drainage between the last set of monitoring wells and the stream bank, we interpret this as evidence that nutrients that were able to infiltrate the buffer were almost completely removed. Coupling this volume with the mean nitrogen and phosphorus concentrations in the field tile suggests an approximate loading reduction of 15 kg of nitrogen and 0.25 kg of dissolved phosphorus. Extending these results beyond dissolved nutrients also indicates near complete removal of sediment and particulate runoff from field tile into the buffer as field tile total suspended solids averaged 10 mg/L, illustrating an additional reduction of 40 total kg of sediment over the study period. And while these sediment removal values are important from a water quality perspective, they may be most important in helping to estimate the maintenance life of the system as a silted in buffer would be undoubtedly reduced in processing potential.

Not surprisingly, the buffer was most effective when nutrient inputs were at their highest. These high levels took place in the earliest part of this study monitoring period (summer 2018) coinciding with nutrient applications and tapering off throughout the year. Unfortunately, given the high levels of precipitation in spring 2019, nutrient applications were delayed, thus, early spring applications for the next year’s crop at the end of the monitoring period were not captured in this study as they had not yet occurred. One important caveat of this study is that although the ultimate fate of nutrients was not tracked, we assume a combination of physical as well as biological processes such as denitrification or uptake into riparian vegetation acted as the primary transformation mechanisms for tile borne nutrients (Groh et al., 2019). And while, in the case of nitrogen removal, denitrification does represent a more desirable path than sequestration in plant material, it should be noted that without a similar pathway that phosphorus in these systems is likely adsorbing to soil particles in buffer as well as stratifying along the surface as nutrient uptake by the plant from the subsurface can concentrate phosphorus into biomass at the surface, thus increasing surface runoff potential of the riparian (Hoffmann et al., 2009) as well as potentially reducing efficacy with age of the system as phosphorus binding sites in the soil are used up. In response to phosphorus stratification potential, studies looking at riparian sequestration of nutrients may incorporate harvesting (e.g., haying) of the buffer vegetation on a periodic basis to combat this pattern and further recycle these nutrients. Future studies in this region of the Midwest should include additional analyses, such as soil testing before and after buffer operation to assess storage capacity and the potential for release over time, water level monitoring from control wells in areas not subject to infiltration, as well as dye tracing to time infiltration rates more effectively, to work to begin to disentangle the myriad of physical and biological mechanisms that are likely acting to varying degrees in the nutrient reductions seen as a result of these systems.

Contextualizing our results with the few published studies that exist indicate similarities with other estimates of nitrogen removal but differences in phosphorus variation. Specific to nitrogen, while our concentration reduction data is on the higher end of published removal efficiency, our percent tile inflow infiltration rates are much closer to the lower end of this scale where diversion efficiencies have been noted as ranging from 20+ to over 90% (Jaynes et al., 2018; Jaynes & Isenhart, 2019). Other analyses that have explored the potential for dissolved phosphorus removal have largely indicated little or no effect attributable to buffers but also acknowledge that some removal from plant uptake or adsorption onto soil particles is possible and even likely in some systems (Utt, Janyes & Albertsen, 2015). We attribute differences between studies dealing with removal efficiencies and volume processing capabilities to a combination of factors including watershed size, precipitation timing, buffer length, riparian to field elevation differential, as well as soil infiltration potential. All of these factors are known to influence the volume and rate that water may move through soil as well as resulting nutrient reduction potential. Focusing on volume of water, we attribute our reduction relative to other studies as primarily a function of the reduced infiltration rate inherent in clay rich soils in which the buffer was situated rather than the watershed size (i.e., volume of runoff) or length of the buffer (300+ m) which both compare favorably with noted higher infiltration rated settings (Jaynes & Isenhart, 2019).

Given the paucity of these systems in the Lake Erie region, there is still some uncertainty as to the optimal configuration and potential efficacy of implementation on a regionwide scale. Since this system represents one of the first in the region, there is an obvious need for additional monitoring on other field sites around northwest Ohio. In addition, since controlled drainage, by definition, limits runoff as a function of increasing the capacity of field soils, there is a need for some of this monitoring in the region to include paired free-flowing watershed comparisons to couple the buffer mitigation potential with the aforementioned practice. Future systems should continue to follow the design standards outlined in the USDA-NRCS Saturated Buffer Standards (Code 604), which effectively outlines ideal site configurations to maximize loading reduction efficiencies while minimizing on-site maintenance. Ideally, buffer sites should be vegetated with a diverse assemblage of deeply rooted perennials as wide as possible (minimum of 10 m) adjacent to stable stream bank, situated at least 0.75 to 1 m in elevation below an adjacent field area, and comprised of relatively organic soils (~2% or more) capable of holding a steady water table during infiltration events (Tomer et al., 2017; Jaynes et al., 2018).

Regardless of these limitations and few existing saturated buffers in the region, a recent feasibility study in the Midwest indicated that Ohio contains at least 6,500+ km of stream bank adjacent tile drained agricultural land that fit within these ideal parameters (Chandrasoma, Christianson & Christianson, 2019). Given the numerous published cost estimates of installing this practice that fall at or below $5,000 USD as well as the practicality of utilizing existing or developing riparian buffer strips, this practice seems poised to expand. With a lifespan at least 15–20 years with little to no maintenance in ideal settings, this best management practice compares favorably from a cost perspective to other practices and could occupy a potential place in restoration plans for the region (Jaynes & Isenhart, 2014).

Over the past several years there has been a notable increase in interest and development of the necessary background information required to make saturated buffers a reality on a wider scale. And while phosphorus removal efficiencies require additional study, there is strong support that the practice can substantially reduce nitrogen loading. Given the loading reduction goals in northwest Ohio, there is a need for a variety of practices to help curtail nonpoint source runoff in the region. Saturated buffers could represent a contribution to resource management and sustainability.

## Conclusions

Saturated buffers can be implemented as part of the overarching nutrient remediation strategy in the Midwestern United States to reduce nutrient loading from nonpoint source tile runoff. In this study, we demonstrated concentration and volume reductions of tile runoff as a result of routing tile water through the riparian zone. However, while this new and innovative approach was shown to be successful it did not eliminate loading completely and is context specific. Moreover, additional work needs to be done in future studies which addresses the various abiotic and biotic processes primarily responsible for nutrient reduction and identify whether these are site (context) specific to better facilitate projections of efficacy at future sites. Thus, while saturated buffers show promise and should be looked on as part of the solution rather, more monitoring is needed. Perhaps most striking from the monitoring of this project, however, was that over the 12-month study period, controlled drainage was used to redirect nearly 25% of the total tile flow into the riparian zone from a subwatershed in a corn/soybean rotation with near complete reductions of dissolved nitrogen and phosphorus from tile inflows averaging 4.7 and 0.08 mg/L, respectively. This study encapsulates only one area in the Midwest in the Lake Erie basin and should be followed up with additional case studies and ultimately, wider scale meta analyses.

## Supplemental Information

10.7717/peerj.9007/supp-1 Supplemental Information 1 Raw nutrient data.

## Additional Information and Declarations

### Field Study Permissions

The following information was supplied relating to field study approvals (i.e., approving body and any reference numbers): Water samples were collected in accordance with water quality monitoring research conducted under a Great Lakes Restoration Initiative monitoring site located on private property. Verbal land owner permission was obtained. No field permits or collecting permits or ethics committee approvals were required. Prior to the start of the project, permission was obtained from the landowner and operator (Luke Vantilburg, VanTilburg Farms, Mercer County, Ohio) to access the site and monitor water quality associated with conservation practices for the duration of the project.

### Data Availability

The following information was supplied regarding data availability: Raw measurements are available as a Supplemental File.
