# Supplementary material for: On the potential for saturated buffers in northwest Ohio to remediate nutrients from agricultural runoff
Source: PeerJ. 2020 Apr 21;8:e9007. doi: 10.7717/peerj.9007 (PMC7182020; doi:10.7717/peerj.9007)
Supplement: Supplemental Information 1 [file peerj-08-9007-s001.pdf]

Date	Tile NO3	Well 1 NO3	Well 2 NO3	Well 3 NO3	Well 4 NO3	Tile DRP	Well 1 DRP	Well 2 DRP	Well 3 DRP	Well 4 DRP	Tile TSS
6/20/2018	47.2	1.85	2.5	0.12	0.408	0.16	0.035	0.036	0.009	0.014	0.5
6/25/2018	18.5	1.96	2.5	0.185	0.116	0.117	0.015	0.006	0.024	0.006	0.8
6/27/2018	15.6	2.1	1.91	0.135	0.262	0.117	0.001	0.006	0.034	0.029	9.2
8/13/2018	6.33	0.669	1.44	0.219	0.132	0.156	0.042	0.009	0.01	0.03	14.8
8/20/2018	3.75	0.234	1.4	0.212		0.162	0.163	0.113			0.5
8/22/2018	2.16	0.765	0.725	0.103	0.126	0.15	0.001	0.003	0.147	0.012	
8/31/2018	4.86	0.225	1.47	0.075	0.143	0.13	0.001	0.001	0.008	0.017	5.6
9/10/2018	3.97	0.073	0.296	0.11	0.08	0.189	0.01	0.021	0.001	0.011	18
10/8/2018	1.61	0.471	1.14	0.187	0.117	0.048	0.001	0.001	0.005	0.001	15.2
11/2/2018	1.5	0.573	0.697	0.115	0.399	0.05	0.001	0.001	0.001	0.005	
11/16/2018	1.01	0.547	0.762	0.094	0.064	0.049	0.001	0.001	0.001	0.001	8.8
11/29/2018	1.18	0.889	1.37	0.079	0.092	0.023	0.001	0.001	0.001	0.001	2.4
12/6/2018	1.05	0.94	1.24	0.069	0.072	0.014	0.001	0.001	0.001	0.001	4.8
12/19/2018	1.19	0.928	1.08	0.144	0.067	0.021	0.012	0.001	0.001	0.001	18
12/31/2018	1.08	1.29	1.03	0.087	0.09	0.035	0.004	0.001	0.001	0.001	24.8
2/8/2019	2.16	2.26	1.13	0.091	0.064	0.078	0.001	0.001	0.001	0.001	11.2
2/26/2019	0.008	3.89	1.28	0.148	0.074	0.044	0.055	0.006	0.015	0.016	9.6
3/12/2019	2.16	2.46	1.48	0.402	0.371	0.01	0.001	0.01	0.001	0.001	8
3/19/2019	2.13	3.86	1.75	0.095	0.587	0.045	0.001	0.001	0.001	0.001	4.8
3/26/2019	0.144	1.08	0.94	0.069	0.054	0.024	0.001	0.001	0.001	0.001	11.6
4/2/2019	3.17	3.34	1.68	0.095	0.078	0.027	0.001	0.004	0.017	0.001	3.2
4/16/2019	2.45	2.95	1.63	0.098	0.083	0.047	0.021	0.021	0.036	0.01	12.8
4/23/2019	1.61	4.59	1.83	0.08	0.067	0.016	0.001	0.001	0.012	0.024	4.4
4/29/2019	1.3	5.59	1.66	0.081	0.139	0.078	0.022	0.018	0.012	0.034	0.5
5/6/2019	1.27	5.88	1.72	0.085	0.264	0.048	0.03	0.035			18
5/13/2019	0.949	2.7	1.31	0.059	0.302	0.104	0.03	0.016	0.006	0.018	19.2
5/20/2019	1.12	5.63	1.73	0.047	0.037	0.114	0.031	0.027	0.006	0.018	28.4
5/27/2019	0.605	3.59	1.31	0.054	0.023	0.081	0.001	0.034	0.009	0.017	14
6/24/2019	7.32	1.64	0.851	0.135	0.126	0.012	0.007	0.018	0.032	0.03	16.8
